# The Effects of Cysteamine Zinc Supplementation on Blood Hormones, Intestinal Flora, and Metabolites in Lactating Mares

**DOI:** 10.3390/ani16142239

**Published:** 2026-07-19

**Authors:** Fan Yang, Xiaobin Li, Xinkui Yao, Jun Meng, Jianwen Wang

**Affiliations:** Xinjiang Key Laboratory of Horse Breeding and Exercise Physiology, College of Animal Science, Xinjiang Agricultural University, Urumqi 830052, China; yangfan312au@163.com (F.Y.);

**Keywords:** lactating mare, cysteamine zinc, hormones, plasma metabolomics

## Abstract

This study aimed to investigate the effects of cysteamine zinc (CS-Zn) supplementation on blood biochemical indices, blood hormones, fecal fermentation, fecal microbial diversity, and blood metabolites in lactating mares. Blood samples were collected via jugular venipuncture, and fecal samples were collected via rectal sampling before morning tethering on days 30, 60, and 90 of the trial. Fecal samples were analyzed for volatile fatty acids (VFAs) and microbial diversity, while changes in blood biochemical indices, blood hormones, and blood non-targeted metabolomics were also analyzed. The results showed that compared with the control group, the test group exhibited significant increases in total protein, globulin, urea, and glucose in the blood (*p* < 0.05); growth hormone (GH) levels significantly increased, while somatostatin levels significantly decreased (*p* < 0.05); and concentrations of all VFAs in feces significantly increased (*p* < 0.05). Simultaneously, CS-Zn supplementation significantly reduced the relative abundance of *Family_XIII_UCG-001*, *Monoglobus*, and *UCG-007* at the genus level. Metabolomics analysis revealed that differentially expressed metabolites were enriched in pathways such as neuroactive ligand–receptor interaction, phenylalanine, tyrosine and tryptophan biosynthesis, and dopaminergic synapse, with significant upregulation of L-tyrosine and dopamine in the blood. These findings indicate that CS-Zn supplementation, on the one hand, can enhance pathways such as glucose metabolism, tyrosine metabolism, and bile secretion by depleting somatostatin and increasing growth hormone levels, and on the other hand, it can improve energy metabolism in mares by altering intestinal microbiota structure and enhancing fiber fermentation efficiency.

## Simple Summary

This study evaluated the effects of cysteamine zinc supplementation on lactating mares. Twelve mares were divided into two groups and received daily doses of 0 and 7 mg/kg body weight of cysteamine zinc, respectively. The results showed that the 7 mg/kg dose significantly depleted somatostatin levels, increased growth hormone levels, and improved fiber fermentation efficiency in the intestines of the horses by enhancing glucose metabolism, tyrosine metabolism, and bile secretion metabolism, while altering the gut microbiota structure.

## 1. Introduction

Milk production is a complex physiological process whose material foundation is entirely derived from nutrients ingested and utilized by the mother. The lactation performance of mares is not only directly related to the health and growth rate of foals but also represents an important manifestation of economic benefits in the horse breeding industry [1]. However, lactation performance is subject to synergistic regulation by multiple factors, including the endocrine system (such as blood hormone levels including prolactin and growth hormone) [2], the intestinal microecological environment (affecting digestion, absorption, and metabolism of nutrients) [3], and genetic factors [4]. Hormone levels are crucial for mammary gland development, lactation initiation, and maintenance in lactating animals; for instance, growth hormone can influence mammary development, milk yield, and metabolic homeostasis in animals during lactation. Shari et al. [5] found in ruminant studies that growth hormone can directly act on mammary cells, stimulating their proliferation and differentiation, thereby providing a structural foundation for milk synthesis and secretion. Prolactin and growth hormone promote fat mobilization and increase glucose production, ensuring adequate glucose supply to the mammary gland as a substrate for lactose synthesis, thereby enhancing milk yield [6]. Cortisol regulates mammary cell gene expression, influencing the synthesis of milk proteins (such as casein), lactose, and milk fat [7]. Endocrine regulation during the lactation period has a critical impact on lactation performance and reproductive function in mares. However, safe and efficient nutritional regulators specifically for lactating mares remain relatively scarce.

Cysteamine zinc, formed by chelation of cysteamine with zinc, possesses the biological activity of cysteamine while overcoming its disadvantages of easy oxidation, poor stability, and poor palatability during feeding, making it a highly efficient nutritional regulator [8]. Cysteamine has been confirmed to deplete somatostatin in the body, thereby relieving its inhibition of anabolic hormones such as growth hormone and insulin-like growth factor-1, indirectly promoting protein synthesis and body growth [9]. Han [10] found that oral administration of cysteamine in sheep increased plasma GH and IGF-1 levels. Wang et al. [11] demonstrated that supplementation with cysteamine zinc sulfate in mid-lactation Holstein dairy cows significantly reduced plasma somatostatin levels and increased growth hormone content. Jia-dong et al. [12] proved that cysteamine supplementation in dairy cows significantly increased blood T_3_, T_4_, and insulin levels, thereby improving milk yield and milk composition. Currently, cysteamine is mostly studied and applied in dairy cows, with results consistently proving its positive effects on milk production performance and hormone levels. However, no studies have yet combined the hormonal changes induced by cysteamine zinc with metabolomics to deeply understand the interaction between hormonal changes and other metabolic pathways.

Therefore, this experiment selected lactating Yili mares as the research subject to investigate the effects of CS-Zn supplementation on blood hormones, fecal fermentation, and intestinal microbiota under consistent feeding conditions. Additionally, metabolomics technology was employed to analyze changes in blood metabolites. This study aims to elucidate the metabolic pathways through which cysteamine zinc affects hormone levels, providing new theoretical foundations and practical guidance for endocrine regulation in horses.

## 2. Materials and Methods

### 2.1. Experimental Design and Treatments

This feeding trial was conducted from June to October 2023 at the Kuder Pasture of Zhaosu Horse Farm, Zhaosu County, Ili Kazakh Autonomous Prefecture, lasting for 100 days (10-day pre-feeding period and 90-day formal feeding period). All experimental treatments and procedures were approved by the Experimental Animal Welfare and Ethics Committee of Xinjiang Agricultural University (approval no.: 2021092).

The lactation performance data in this study were derived from the author’s previous research [13]. In the previous study, test groups with supplementation doses of 3 mg/kg·BW and 5 mg/kg·BW were also established. However, it was found that at 90 days of the trial, compared to the other dose groups, mares in the 7 mg/kg·BW group exhibited the highest milk yield, with milk protein percentage, milk fat percentage, and lactose content all significantly higher than those of the control group. Therefore, the subsequent detection and analysis of fecal microbiota and milk metabolites in this study were conducted only for the 0 mg/kg·BW group and the 7 mg/kg·BW group.

This experiment included 12 healthy lactating mares (used primarily for milk production) with similar foaling dates (20 May 2023–30 May 2023) and an average body weight of 395.50 ± 28.60 kg. Randomization was performed using a random number generator. The mares were equally divided into a control group and a treatment group, with 6 animals in each group. No blinding was implemented in this study; animal husbandry staff and laboratory analysts were aware of the group allocation throughout the experiment and data statistical analysis. Under identical grazing conditions (same grazing time, watering time, milking time, and grazing pasture), mares in the control group were supplemented daily with 0 mg/kg·BW CS-Zn (empty capsules), while those in the test group were supplemented with 7 mg/kg·BW. The supplementation dosage was referenced from the study by Wang et al. [11].

According to the mare breeding model, a semi mother–foal integrated experimental design was adopted. During the trial period, mares and foals were driven from the grazing pasture to the milking pasture daily at 08:30. The corresponding dose of CS-Zn for each group was placed into size 00 edible glutinous rice capsules (length, 2.3 cm; diameter, 0.8 cm), which were then administered orally to the mares, ensuring capsule ingestion. At 09:00, mares were separated from their foals. The separation period served as milking time. All mares were tethered for 8 h daily during daytime (09:00–17:00), during which they were isolated from their foals and natural nursing was prohibited. This husbandry treatment was uniformly applied to all experimental mares. Mother–foal separation and prolonged tethering represent relatively intensive husbandry interventions that can easily induce stress responses in lactating mares. The measurement of cortisol indices in this study was specifically designed to assess the physiological changes resulting from such stress. Mares were milked every 2 h; after the final milking at 17:00, the mares were reunited with their foals and returned to the grazing pasture. For the remaining 16 h, mares and foals grazed together freely, and foals were allowed to nurse ad libitum. Throughout the entire trial, mares had free access to feed and water.

### 2.2. Pasture, Plasma, and Fecal Sampling

During days 28–30 of each 30-day period throughout the trial period, fecal samples were collected from the horses using the rectal sampling method, and pasture samples were collected by cutting fresh forage to simulate the grazing habits of horses during pasture feeding. Fecal and pasture samples were immediately stored at −20 °C, then thawed at room temperature and mixed. These samples were used for the determination of routine nutritional components. The dry matter intake of pasture forage was calculated using the acid-insoluble ash (AIA) method, following Song et al. [14].

The calculation formula was:

$$TDI=\;\frac{F\times a}{b}$$

where TDI is the trial horse dry matter intake (kg·day^−1^·horse^−1^); *F* is the daily fecal dry matter excretion (kg·day^−1^·horse^−1^); *a* is the AIA content in fecal dry matter (%); and *b* is the AIA content in consumed pasture dry matter (%). After calculation, the average dry matter intake of the trial horses was 12.98 ± 2.25 kg. The pasture species mainly included orchard grass (*Dactylis glomerata*), smooth brome (*Bromus inermis*), prairie sedge (*Carex liparocarpos*), clover (*Trifolium*), prairie sage (*Phlomoides pratensis*), and various weeds. The nutritional levels of the mixed pasture in the grazing pasture during the trial period are shown in Table 1.

On days 0, 30, 60, and 90, 10 mL of blood plasma was collected from the jugular vein of each horse before morning tethering, and 50 mL of feces was collected using the rectal sampling method. Both plasma and fecal samples were frozen at −80 °C for subsequent hormone, metabolite, and microbial analyses.

### 2.3. Blood Biochemical Indices and Hormone Analysis

The plasma indices measured in this experiment included two categories: biochemical indices and hormone indices. Biochemical indices related to protein metabolism, carbohydrate metabolism, and lipid metabolism included total protein (TP), albumin (ALB), globulin (GLB), urea, glucose (Glu), triglycerides (TG), total cholesterol (TCH), total bilirubin (T-Bil), and direct bilirubin (D-Bil). All biochemical detection kits were purchased from Nanjing Jiancheng Bioengineering Institute (Nanjing, China). Hormone indices, including growth hormone (GH), somatostatin (SS), triiodothyronine (T_3_), thyroxine (T_4_), estradiol (E_2_), prolactin (PRL), and cortisol (Cor), were quantified using kits provided by Beijing Huaying Biotechnology Research Institute (Beijing, China). The corresponding catalog numbers were as follows: HY-C0018 (ELISA) for GH, HY-10172 (RIA) for SS, HY-10001 (RIA) for T_3_, HY-10002 (RIA) for T_4_, HY-10029 (IRMA) for E_2_, HY-10026 (IRMA) for PRL, and HY-10062 (RIA) for Cor. According to the manufacturer’s official specifications, the intra-assay coefficient of variation (CV) and inter-assay CV for the GH, SS, T_3_, T_4_, E_2_, and Cor kits were less than 10% and 15%, respectively, while the intra-assay CV and inter-assay CV for the PRL kit were less than 5.4% and 8.5%, respectively. The minimum detection limits for GH, SS, T_3_, T_4_, E_2_, PRL, and Cor were 0.1 ng/mL, 10 pg/mL, 0.25 ng/mL, 3 ng/mL, 5 pg/mL, 0.4 ng/mL, and 1 ng/mL, respectively. All sample processing and index detection procedures were strictly performed in accordance with the standard protocols provided by the respective kit manufacturers.

### 2.4. Fecal pH and VFA Analysis

Ten grams of fresh fecal sample were mixed with 10 mL of ultrapure water, vortexed for 3 min, filtered through four layers of gauze, and the pH of each sample was measured using a calibrated pH meter (FiveEasy 22-Meter, Mettler-Toledo International Trade (Shanghai) Co., Ltd., Shanghai, China).

Volatile fatty acids (VFAs) in the fecal extracts were determined using a Shimadzu GC-2010 gas chromatograph equipped with a Stabilwax column (Kyoto, Japan), with 4-methylvaleric acid as the internal standard. Chromatographic conditions were as follows: injection port temperature, 230 °C; column oven initial temperature, 55 °C; ramped at 13 °C/min to 200 °C and held for 0.5 min; FID detector temperature, 240 °C.

Sample processing and determination: 10 g of fecal sample was mixed with 10 mL ultrapure water, vortexed for 3 min, filtered through four layers of gauze, and centrifuged at 10,000 rpm for 10 min. The supernatant was collected, store at 4 °C for 12 h, then centrifuged at 15,000 rpm for 15 min. One milliliter of the filtrate was centrifuged at 15,000 rpm for 5 min, and 0.5 mL of the supernatant was transferred to a 1.5 mL centrifuge tube. Then 0.5 mL of 10% TCA and 0.1 mL of 40 mmol 4-methylvaleric acid were added, mixed thoroughly with a vortex mixer, allowed to stand for 20 min, and centrifuged at 20,000× *g* for 15 min. One milliliter of the supernatant was transferred to a sample vial. A 0.5 μL aliquot was injected for analysis to obtain the VFA chromatogram. The regression equation was established using the VFA concentration ratio as the y-axis and the peak height ratio as the x-axis by the internal standard method to calculate the VFA content in the samples.

### 2.5. Fecal 16S Analysis

Fecal samples were removed from liquid nitrogen and thawed, and genomic DNA was extracted using the hexadecyltrimethylammonium bromide (CTAB) method. Purity and concentration were detected by 0.8% agarose gel electrophoresis and diluted to 1 ng/μL for standby use. The diluted genomic DNA was used as a template, and the V3-V4 region-specific primers 341F and 806R of 16S rDNA were used for amplification.

341 F: 5′-CCTAYGGGRBGCASCAG-3′.

806 R: 5′-GGACTACNNGGGTATCTAAT-3′.

The extracted bacterial total DNA and 16S rDNA-V3~V4 region sequence amplification showed correct target band sizes, with total amounts sufficient for more than two library constructions, suitable for subsequent library preparation.

All PCR mixtures contained 15 µL Phusion^®^ High-Fidelity PCR Master Mix (New England Biolabs, Ipswich, MA, USA), 0.2 µM primers, and 10 ng genomic DNA template. The first denaturation was performed at 98 °C for 1 min, followed by 30 cycles at 98 °C (10 s), 50 °C (30 s), and 72 °C (30 s), with a final extension at 72 °C for 5 min.

PCR products were purified with magnetic beads. Equal amounts of PCR products were mixed according to concentration. After thorough mixing, PCR products were detected and target bands were recovered.

Library construction was performed. The constructed library was quantified by Qubit and Q-PCR. After passing quality control, PE250 sequencing was performed on the NovaSeq6000 platform (Illumina, San Diego, CA, USA).

Sample data were split from the raw sequencing data based on Barcode sequences and PCR amplification primer sequences. After removing Barcode and primer sequences, FLASH (Version 1.2.11, http://ccb.jhu.edu/software/FLASH/, accessed on 16 July 2026) was used to assemble reads for each sample. The assembled sequences were designated as Raw Tags. Cutadapt software (Version: 5.2) was then used to match reverse primer sequences and trim remaining sequences to prevent interference with subsequent analysis. The fastp software (Version 0.23.1) was used for strict filtering of assembled Raw Tags to obtain high-quality Clean Tags data. The resulting Tags required removal of chimeric sequences. Tags sequences were compared with species annotation databases (Silva database https://www.arb-silva.de/, accessed on 16 July 2026, for 16S/18S) to detect chimeric sequences, which were ultimately removed to obtain final Effective Tags.

### 2.6. Plasma Non-Targeted Metabolomics Analysis

Exactly 100 μL of sample was transferred to a 1.5 mL centrifuge tube. Four hundred microliters of extraction solvent (methanol:acetonitrile = 1:1 (*v*:*v*)) containing four internal standards (including L-2-chlorophenylalanine (0.02 mg/mL)) was added. After vortexing for 30 s, low-temperature ultrasonic extraction was performed for 30 min (5 °C, 40 kHz). Samples were placed at −20 °C for 30 min then centrifuged for 15 min (13,000 *g*, 4 °C). The supernatant was collected, dried under nitrogen gas, and reconstituted with 120 μL of reconstitution solution (acetonitrile:water = 1:1). After vortexing for 30 s, low-temperature ultrasonic extraction was performed for 5 min (5 °C, 40 kHz). After centrifugation for 10 min (13,000 *g*, 4 °C), the supernatant was transferred to injection vials with inserts for analysis. Additionally, 20 µL of supernatant from each sample was mixed to serve as a quality control sample.

The LC-MS analysis instrument platform was the Thermo Scientific UHPLC-Exploris240 ultra-high-performance liquid chromatography coupled with Fourier transform mass spectrometry system (Waltham, MA, USA). Chromatographic conditions: column was ACQUITY UPLC HSS T_3_ (100 mm × 2.1 mm i.d., 1.8 µm; Waters, Milford, CT, USA); mobile phase A was 95% water + 5% acetonitrile (containing 0.1% formic acid); mobile phase B was 47.5% acetonitrile + 47.5% isopropanol + 5% water (containing 0.1% formic acid); injection volume was 3 μL; and column temperature was 40 °C.

Mass spectrometry conditions: Samples were ionized by electrospray ionization, and mass spectrometry signals were collected in both positive and negative ion scan modes. Specific parameters are shown in Table 2.

### 2.7. Data Analysis

Blood biochemical indices and hormone levels in mares were preprocessed using Excel 2021, followed by two-way ANOVA for time effects (date) and treatment effects (trt) using SPSS 26.0 software. Duncan’s method was used for multiple comparisons between groups. *p* < 0.05 indicates significant difference, *p* < 0.01 indicates extremely significant difference, and *p* > 0.05 indicates no significant difference.

The raw microbial sequencing reads underwent quality filtering, trimming, denoising, and chimera removal using the DADA2 plugin in QIIME2 (Version: 2025.10) to obtain high-quality effective Clean Tags. Qualified clean sequences were further clustered into operational taxonomic units (OTUs) at a 97% sequence similarity threshold. Taxonomic classification of representative OTU sequences was performed using the Silva 138.1 reference database in QIIME2 software for subsequent microbial diversity analysis. Chao1, Shannon, and other indices were calculated for alpha diversity analysis; Bray–Curtis distance matrices were used for principal component analysis (PCA) to characterize beta diversity; *t*-tests were used to calculate relative abundances at the phylum, genus, and species levels; LEfSe analysis was conducted using the LEfSe algorithm; and functional prediction analysis was performed using the Tax4Fun tool (Version:1.1.5).

Metabolomics raw data were imported into the metabolomics processing software Progenesis QI v3.0 (Waters Corporation, Milford, CT, USA) for baseline filtering, peak identification, integration, retention time correction, peak alignment, etc., to finally obtain a data matrix containing retention time, mass-to-charge ratio, and peak intensity information. Subsequently, feature peak database searching and identification were performed using this software. MS and MS/MS mass spectrometry information was matched with metabolite databases. MS mass error was set to less than 10 ppm, and metabolites were identified based on secondary mass spectrometry matching scores. Main databases included https://pubchem.ncbi.nlm.nih.gov/, accessed on 16 July 2026, https://www.ebi.ac.uk/chebi/, accessed on 16 July 2026, and other mainstream public databases. The data were transformed, followed by principal component analysis (PCA) and partial least squares discriminant analysis (PLS-DA), to calculate the variable importance in projection (VIP) value for each metabolite. PCA was used exclusively for exploratory data visualization and not for statistical inference. Univariate analysis was performed using a *t*-test to calculate the statistical significance (*p*-value) of each metabolite between the two groups, and the fold change (FC) was calculated. Differential metabolites were selected based on the following criteria: VIP > 1, *p* < 0.05, and FC ≥ 2 or FC ≤ 0.5.

## 3. Results

### 3.1. Blood Biochemical Indices

Table 3 shows the effect of cysteamine zinc on blood biochemical indicators. Compared with the control group, CS-Zn intake significantly altered some plasma indices. Total protein, globulin, and urea increased in the test group mares (*p* < 0.05), while glucose increased by 10.25% (*p* < 0.01). When time was analyzed as the main influencing factor, TP, GLB, UREA, Glu, and total bilirubin (T-Bil-V) all showed *p*-values <0.05.

### 3.2. Blood Hormone Levels

Table 4 shows the effect of cysteamine zinc on blood hormones. Growth hormone (GH) was significantly affected by the interaction between CS-Zn supplementation and duration (*p* < 0.05). CS-Zn supplementation did not significantly alter overall levels of thyroxine (T_4_) and triiodothyronine (T_3_). Growth hormone (GH) significantly increased in the test group (*p* < 0.01), while somatostatin (S.S) significantly decreased (*p* < 0.05). When time was analyzed as the main influencing factor, supplementation time had extremely significant effects on each measured hormone (*p* < 0.01).

### 3.3. Fecal pH and VFA

Table 5 shows the effects of cysteamine zinc on fecal pH and VFAs. After CS-Zn supplementation, there was no difference in fecal pH between groups (*p* > 0.05), while total VFA (TVFA) and individual VFAs all showed significant changes. Compared with the control group, the test group showed significantly higher TVFA concentration and all measured VFA concentrations (*p* < 0.01). Supplementation time significantly affected fecal pH and butyrate content (*p* < 0.05).

### 3.4. Fecal 16S Analysis

**Figure 1:**
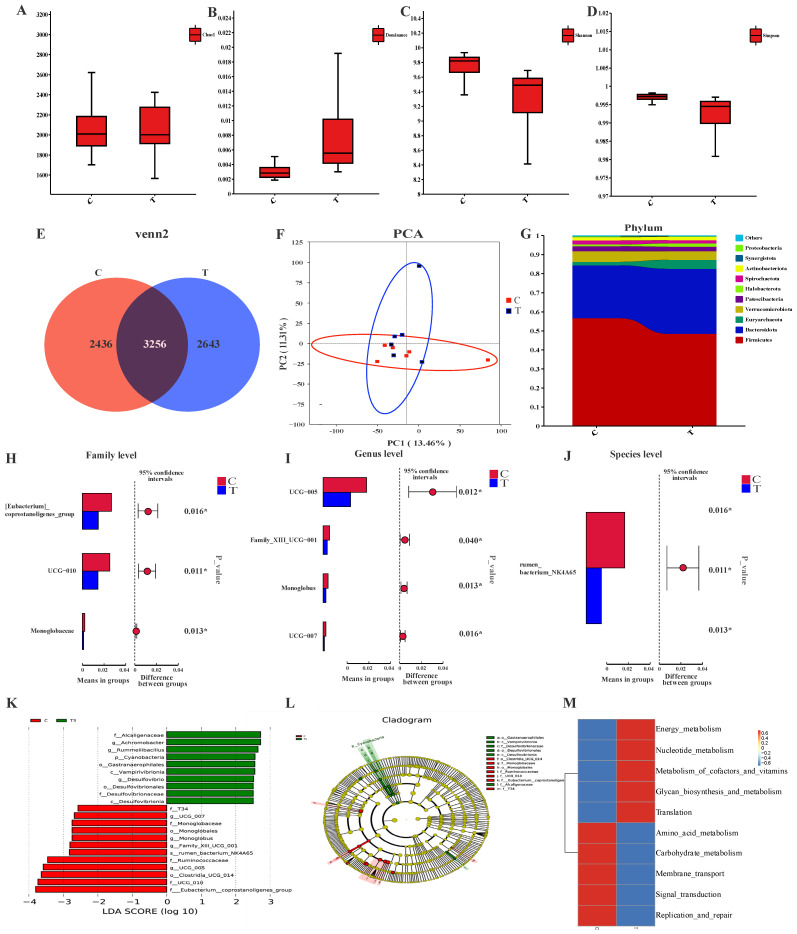
The effect of cysteamine zinc on fecal microbiota. (**A**), (**B**), (**C**), and (**D**) are bar charts of the Chao1, Dominance, Shannon, and Simpson indices, respectively; (**E**) is a Venn diagram; (**F**) is a PCA plot; (**G**) is a bar chart of relative species abundance at the phylum level; (**H**), (**I**), and (**J**) are bar charts of differential bacteria at the family, genus, and species levels, respectively; (**K**,**L**) are LEfSe analyses; (**M**) is Tax4Fun functional prediction. * indicates statistically significant difference between the two groups (*p* < 0.05).

Figure 1A–D show the Chao1, Dominance, Shannon, and Simpson indices in alpha diversity (C = control group; T = test group), respectively; the results indicate that CS-Zn supplementation did not affect fecal alpha diversity. Figure 1E presents a Venn diagram, in which the purple section shows 3256 OTUs shared between the two groups; the control group had 2436 unique OTUs (orange), and the test group had 2643 unique OTUs. Figure 1F shows PCA, with results indicating that inter-group similarity between control and test group samples was not well distinguished. Figure 1G is a bar chart of relative species abundance at the phylum level, showing that Firmicutes, Bacteroidetes, and Verrucomicrobia were the dominant phyla. Figure 1H–J show bacteria with significantly different relative abundances at the family, genus, and species levels, respectively. At the family level, CS-Zn supplementation significantly reduced the relative abundance of [Eubacterium]_coprostanoligenes_group, UCG-010, and Monoglobaceae in the test group mares (Figure 1H). At the genus level, CS-Zn supplementation significantly reduced the relative abundance of UCG-005, Family_XIII_UCG-001, Monoglobus, and UCG-007 in the test group mares (Figure 1I). At the species level, CS-Zn supplementation significantly reduced the relative abundance of rumen_bacterium_NK4A65 in the test group mares (Figure 1J). Figure 1K,L show LEfSe analysis, identifying 22 differentially abundant biomarkers between the control and test groups. Figure 1M presents Tax4Fun-based microbial community functional analysis, revealing the clustering results of predicted fecal microbial functions between the control and test groups. The control group microbiota mainly clustered in amino acid metabolism, carbohydrate metabolism, membrane transport, signal transduction, and replication and repair functions. The test group microbiota mainly clustered in energy metabolism, nucleotide metabolism, metabolism of cofactors and vitamins, glycan biosynthesis and metabolism, and translation functions.

### 3.5. Blood Non-Targeted Metabolomics Analysis

**Figure 2:**
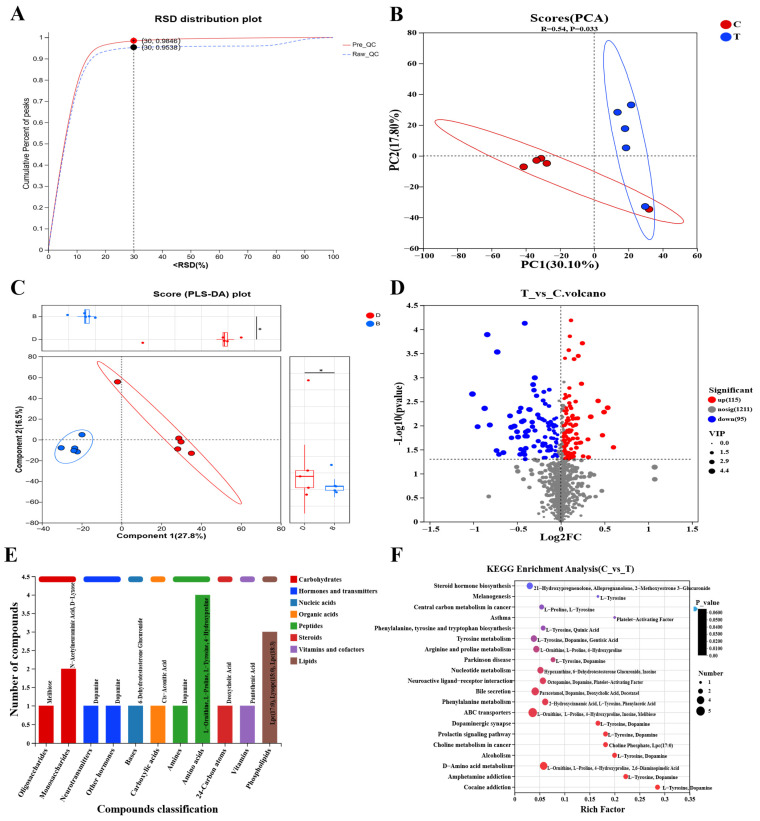
Effects of cysteamine zinc on blood metabolites. (**A**) is the RSD distribution plot; (**B**) is the PCA plot; (**C**) is the PLS-DA plot; (**D**) is the metabolite volcano plot; (**E**) is the Compounds classification plot; (**F**) is the KEGG pathway enrichment analysis plot. * indicates statistically significant difference between the two groups (*p* < 0.05).

Figure 2A shows the RSD distribution analysis. The results indicate that when the abscissa RSD < 30%, the cumulative proportion of peaks exceeded 0.7, demonstrating good stability throughout the detection process, high data quality, and suitability for subsequent metabolite detection and analysis. Figure 2B presents the principal component analysis (PCA), where PC1 explained 30.10% of the total variation and PC2 explained 17.80%; the two principal components collectively contributed to 47.90% of the sample variation. The control group C (red) and treatment group T (blue) samples exhibited a relatively obvious separation trend in elliptical confidence intervals, indicating significant differences in metabolic profiles between groups C and T, and that CS-Zn supplementation had significant effects on mare blood metabolites. Figure 2C shows the PLS-DA discriminant analysis, where Component 1 explained 27.8% of the variation and Component 2 explained 21.63%; the two principal components cumulatively accounted for nearly 50% of the sample variation. The confidence ellipses of groups C and T were completely separated, with high aggregation of replicate samples within each group. The accompanying box plots on the right side intuitively verified a significant overall metabolic shift between the two groups, further confirming that the treatment intervention induced specific metabolic remodeling, and that the model could effectively distinguish between the two sample groups, making it suitable for differential metabolite screening. Figure 2D presents the volcano plot, showing that a total of 1211 metabolites were identified in the two groups of mare blood samples, including 115 significantly upregulated metabolites and 95 significantly downregulated metabolites. Subsequently, the top 15 differential metabolites detected were classified according to KEGG compound classification based on *p*-values (Figure 2E). Based on the biological functions of metabolites, they were mainly divided into carbohydrates, hormones and transmitters, nucleic acids, organic acids, peptides, steroids, vitamins and cofactors, and lipids. Figure 2F shows the results of screening the top 20 metabolic pathways after KEGG pathway enrichment of differential metabolites. L-Tyrosine (FC = 1.02, *p* = 0.016) and dopamine (FC = 1.06, *p* = 0.014) jointly participated in the cocaine addiction, amphetamine addiction, alcoholism, prolactin signaling, Parkinson’s disease, and tyrosine metabolism pathways (Figure 2F). The D-amino acid metabolism, ABC transporter, phenylalanine metabolism, bile secretion, neuroactive ligand–receptor interaction, nucleotide metabolism, arginine and proline metabolism, tyrosine metabolism, and steroid hormone biosynthesis pathways all had three or more differential metabolites participating.

## 4. Discussion

Blood biochemical indices can indirectly reflect digestive efficiency, endocrine status, and overall health [15]. In this study, cysteamine zinc supplementation increased plasma concentrations of total protein, glucose, and urea in a dose-dependent manner, while triglycerides and total cholesterol remained unchanged. These results indicate that CS-Zn supplementation affected nutrient digestion and absorption to a certain extent. Cysteamine is a naturally occurring growth promoter in monogastric and ruminant species, capable of reducing somatostatin (S.S) concentrations in the gastrointestinal tract, pancreas, plasma, and hypothalamus. It increases visceral blood flow and portal network absorption, thereby improving feed conversion ratio, growth rate, and lean deposition [16]. Zongzhu [17] reported similar findings in sheep: cysteamine significantly improved apparent digestibility of dry matter and crude protein, and increased microbial protein synthesis in the rumen. The somatostatin-depleting effect of cysteamine also upregulates digestive enzymes in the gastrointestinal tract, further increasing nutrient utilization [18]. Therefore, we speculate that cysteamine zinc may regulate the digestive and metabolic levels of horses by altering endocrine balance or the intestinal microbiome.

In previous studies, exogenous cysteamine hydrochloride (CSH) increased circulating concentrations of growth hormone (GH), thyroid hormones, insulin, glucose, IGF-I/-II, gastrin, and prolactin in multiple species [10,12,19], emphasizing its core role as an endocrine regulator. Bousquet et al. [20] demonstrated that cysteamine hydrochloride (CSH) easily crosses the blood–brain barrier, inhibiting somatostatin (S.S) synthesis and secretion in the hypothalamus. Moreover, cysteamine supplementation in pigs, goats, sheep, and cattle can increase GH secretion [21,22]. Growth hormone release is normally limited by the opposing actions of growth hormone-releasing hormone (GHRH) and S.S; the thiol and amino groups of cysteamine reduce S.S disulfide bonds, alter its tertiary structure, and weaken its inhibitory potency, thereby relieving the inhibition of GH release [23,24,25]. Studies in rat models further confirmed that cysteamine can specifically deplete endogenous somatostatin and significantly upregulate GHRH mRNA levels in the hypothalamus [26]. The elevated GH and reduced S.S observed in this study are similar to previous research results. Thyroid hormones control cellular energy supply and biochemical pathways for milk synthesis [27]. This study showed that T_3_ and T_4_ levels did not significantly differ after CS-Zn supplementation, which differs from the results of Shi et al. [28] and Yang et al. [29], who reported increased plasma T_3_ and T_4_ contents in piglets receiving CSH. This difference may reflect species-specific or breed-related differences in thyroid hormone action. This study monitored plasma prolactin, cortisol, and estradiol, but these hormones were not associated with CS-Zn supplementation, indicating that under current experimental conditions, their regulatory pathways remained undisturbed. Finally, our hormone results showed that each measured endocrine parameter was significantly affected by supplementation time. Wang et al. [11] reported that dairy cows required at least 30 days of oral cysteamine administration before differences in growth hormone and milk yield-related indices were observed. Additionally, Sagar et al. [30] indicated that cysteamine-induced somatostatin depletion is reversible; in rats, somatostatin levels dropped sharply within 2–4 h after a single cysteamine injection, but sustained depletion required more than one week of administration. Blood hormone data suggest that long-term, continuous CS-Zn supplementation is meaningful for maintaining the hormonal environment required for sustained improvement of mare lactation performance.

In this study, CS-Zn supplementation significantly increased fecal VFA concentrations, but fecal pH did not differ significantly. These data indicate that cysteamine zinc altered the energy metabolism of food in the gastrointestinal tract. Intestinal pH is a key determinant of digestive enzyme activity. In the horse intestine, pH is primarily driven by lactic acid and volatile fatty acids (VFAs) produced by cecal and colonic microbial fermentation. Microorganisms first ferment dietary residues into lactic acid, which is then converted to VFAs. These VFAs are absorbed by the intestinal wall, stimulate enteroendocrine signaling, and provide systemic energy [31,32]. This effect may be mediated by elevated growth hormone (GH), which is known to improve digestion and utilization of protein, fat, carbohydrates, and minerals [33,34], consistent with the higher GH concentrations observed here. Previous studies have shown that cysteamine alters hormones regulating smooth muscle, including increased triiodothyronine [12], thereby prolonging mean retention time of digesta [9], allowing for more complete nutrient absorption. Similarly, Besong et al. [35] reported that dietary cysteamine increased acetate, propionate, and total VFA concentrations in bulls. Therefore, we speculate that cysteamine zinc alters the hindgut microbiota of supplemented mares, enriching taxa involved in carbohydrate fermentation, thereby increasing VFA and TVFA contents in the test group mares. Mare lactation requires substantial energy, and volatile fatty acids (VFAs) produced by hindgut fermentation are the main source of this energy. The elevated individual and total VFA concentrations observed in our study reflect increased milk yield and milk fat percentage in supplemented mares, highlighting the critical role of microbial metabolites in supporting lactation performance.

Regarding intestinal microbiota, Jun [36] and Zikun [37] found in studies on cashmere goats and buffalo that cysteamine did not change rumen bacterial or fungal alpha diversity. This study is similar to the results of Jun and Zikun. However, with CS-Zn supplementation, the relative abundance of Firmicutes in feces decreased, while that of Bacteroidetes increased. In non-ruminants, fiber fermentation occurs almost exclusively in the hindgut (cecum and colon), and the composition and abundance of microbial communities directly determine fiber degradation efficiency and subsequent energy supply [38]. The surge in Bacteroidetes paralleled the significant increase in fecal VFA concentrations. In horse studies, Firmicutes, Bacteroidetes, Verrucomicrobia, and Actinobacteria have consistently been the dominant phyla in the gastrointestinal and fecal microbiomes [39,40], with Firmicutes and Bacteroidetes usually predominating. Firmicutes specialize in degrading complex carbohydrates; generally, animals with high carbohydrate diets have more abundant Firmicutes in their digestive tracts [41]. In contrast, Bacteroidetes excel at breaking down resistant starch and dietary fiber into formate, acetate, lactate, propionate, and other VFAs to supply energy for mare lactation activities, while improving the intestinal environment and nutritional status [42]. Therefore, we speculate that the control group microbiota was primarily involved in carbohydrate metabolism, whereas the test group microbiota was mainly engaged in energy metabolism and glycan biosynthesis metabolism, which is also consistent with the results from the functional prediction (Tax4Fun functional prediction is merely an inferential result rather than direct functional detection). Furthermore, this study also observed significant reduction of [Eubacterium]_coprostanoligenes_group at the family level and improved mare milk fat levels. Wei et al. [43] found in mouse studies that the abundance of [Eubacterium]_coprostanoligenes_group is related to host lipid metabolism; the more intense the lipid metabolism, the lower its relative abundance. This is similar to our study results, indicating that CS-Zn improved lipid metabolism in mares, thereby reducing the abundance of [Eubacterium]_coprostanoligenes_group. Meanwhile, CS-Zn supplementation also reduced the relative abundance of Monoglobaceae. Gámez-Macías [44] found in studies on elderly individuals that increased immunity may be associated with reduced relative abundance of Monoglobaceae in the intestine. The decrease in Monoglobaceae may have enhanced the immunity of the mares. Therefore, we believe that cysteamine zinc has a dual mode of action: on one hand, it alters the endocrine environment controlling nutrient allocation (such as growth hormone, somatostatin); on the other hand, it alters the relative abundance of intestinal microbiota, thereby increasing VFA production and improving energy metabolism efficiency.

Finally, non-targeted metabolomics detection of horse blood showed that among differential metabolites, L-tyrosine and dopamine were significantly upregulated, and significantly enriched in pathways such as neuroactive ligand–receptor interaction, phenylalanine, tyrosine and tryptophan biosynthesis, and dopaminergic synapse. L-tyrosine is a key precursor for synthesizing catecholamine neurotransmitters such as dopamine and norepinephrine. In Souza’s [45] research, it was shown that in the process of L-tyrosine synthesizing dopamine, tyrosine hydroxylase (TH) is the key rate-limiting enzyme, and its activity directly determines neurotransmitter content, representing the most critical step in catecholamine production. In the study by Villalobos et al. [46], specific knockout of GH receptors (D2) in TH neurons significantly increased GH concentration in mice, suggesting that the elevated GH levels in mares caused by CS-Zn in this study may be mediated by activation of these TH neurons, thereby regulating tyrosine hydroxylase activity, altering tyrosine metabolism and TH activity, and thus changing GH levels. The enrichment of tyrosine metabolism and dopaminergic synapse pathways in metabolomics represents the manifestation of this systemic regulation at the blood metabolite level. The arginine and proline metabolism pathway also changed in this study. Córdoba et al. [47] showed that arginine can inhibit somatostatin secretion through multiple mechanisms including affecting ATP/ADP ratios, activating G protein-coupled receptors, and generating nitric oxide. Therefore, the reduced S.S levels in mare blood may be related to arginine metabolism. Additionally, the bile metabolism pathway also changed significantly in this study. Magnusson et al. [48] showed that in humans, somatostatin can inhibit bile formation, mainly by reducing canalicular bile flow. At physiological doses, somatostatin can rapidly reduce bile flow (by approximately 30%) and bile acid secretion (32–47%) [49]. Bajor et al. [50] showed that bile acids play a key role in lipid digestion and absorption, while changes in intestinal fermentation substrates affect the production of microbial metabolites. Wang et al. [51] found in lamb studies that increased bile acids can significantly increase VFAs produced by intestinal microbiota fermentation, and liver differential metabolites were significantly enriched in the bile secretion pathway, with significant correlations between these metabolites and intestinal VFA (such as propionate, butyrate) content and proportions, similar to our study results. This suggests that after CS-Zn supplementation, the reduced somatostatin levels may have weakened the inhibition of bile secretion, leading to changes in bile secretion and related metabolic processes observed in this study. This may be indirectly related to the elevated fecal volatile fatty acid concentrations observed in this study. The above results should be regarded as testable hypotheses rather than definitive established pathways, and future studies specifically designed for functional validation will be required to confirm these sequential regulatory interactions.

## 5. Conclusions

In summary, supplementation with 7 mg/kg·BW of cysteamine zinc can significantly increase blood total protein, glucose, and growth hormone levels, increase fecal VFA concentrations, alter intestinal microbiota, and significantly upregulate differential metabolites such as L-tyrosine and dopamine, while enriching pathways such as neuroactive ligand–receptor interaction, phenylalanine, tyrosine and tryptophan biosynthesis, and dopaminergic synapse. However, the small sample size in this study limits the generalizability of the results, and all multi-omics analyses only reveal correlations among the microbiota, metabolites, and host phenotypes rather than causal interactions; further functional experiments are required to validate the regulatory mechanisms.

## Figures and Tables

**Table 1:** Nutritional level of forage (measured value after air drying).

Items	Content
DM (%)	91.58
OM (%)	92.64
GE (MJ/kg)	17.64
CP (%)	11.28
EE (%)	2.83
NDF (%)	44.70
ADF (%)	28.31
Ca (%)	0.87
P (%)	0.17

**Table 2:** Mass spectrometry parameters.

Description	Parameter
Scan type (*m*/*z*)	70–1050
Sheath gas flow rate (arb)	60
Aux gas flow rate (arb)	20
Heater temp (°C)	350
Capillary temp (°C)	320
Spray voltage (+) (V)	3400
Spray voltage (−) (V)	−3000
S-Lens RF Level	70
Normalized collision energy (%)	20, 40, 60
Resolution (Full MS)	60,000
Resolution (MS2)	15,000

**Table 3:** Effects of cysteamine zinc on blood biochemical indices.

Items	Control	Test	SEM	*p*-Value		
				Trt	Date	Trt × Date
TP (g/L)	63.44	66.84	0.91	0.012	0.001	0.276
ALB (g/L)	28.81	28.44	0.40	0.509	0.678	0.444
GLB (g/L)	34.53	38.41	0.90	0.005	0.003	0.191
UREA (μmol/L)	6.88	7.53	0.19	0.016	0.026	0.546
Glu (mmol/L)	4.78	5.38	0.10	<0.001	0.041	0.045
TG (mmol/L)	0.28	0.25	0.01	0.164	0.288	0.430
TC (mmol/L)	1.75	1.74	0.04	0.896	0.234	0.305
T-Bil-V (μmol/L)	3.51	3.73	0.79	0.833	0.047	0.319

**Table 4:** Effects of cysteamine zinc on hormones.

Items	Control	Test	SEM	*p*-Value		
				Trt	Date	Trt × Date
T_4_ (ng/mL)	28.59	28.37	0.45	0.728	<0.001	0.808
T_3_ (ng/mL)	0.80	0.76	0.01	0.076	<0.001	0.420
GH (ng/mL)	4.50	6.01	0.27	<0.001	<0.001	<0.190
S.S (pg/mL)	21.41	18.97	0.68	0.017	<0.001	0.331
PRL (uIU/mL)	269.39	258.16	7.32	0.286	<0.001	0.633
COR (ng/mL)	11.51	11.15	0.20	0.208	<0.001	0.356
E_2_ (pg/mL)	34.19	35.24	0.88	0.405	<0.001	0.929

**Table 5:** Effects of cysteamine zinc on fecal pH and VFAs.

Items	Control	Test	SEM	*p*-Value		
				Trt	Date	Trt × Date
pH	6.13	6.11	0.04	0.703	0.017	0.040
TVFA (mmol/L)	26.84	36.87	1.85	<0.001	0.594	<0.001
Acetic acid (mmol/L)	20.03	26.62	1.33	0.001	0.779	<0.001
Propionic acid (mmol/L)	4.19	5.41	0.32	0.013	0.348	0.001
Isobutyric acid (mmol/L)	0.36	0.57	0.04	<0.001	0.295	0.009
Butyric acid (mmol/L)	1.59	3.03	0.19	<0.001	0.016	<0.001
Isovaleric acid (mmol/L)	0.44	0.78	0.06	<0.001	0.434	0.013
Valeric acid (mmol/L)	0.23	0.45	0.03	<0.001	0.347	0.004

## Data Availability

The raw data supporting the conclusions of this article will be made available by the authors on request.

## Abbreviations

The following abbreviations are used in this manuscript:

BW	Body Weight
CS-Zn	Cysteamine Zinc
CSH	Cysteamine hydrochloride
TP	Total Protein
GLB	Globulin
Glu	Glucose
GH	Growth Hormone
T_3_	Triiodothyronine
T_4_	Thyroxine
VFAs	Volatile Fatty Acids
TVFA	Total Volatile Fatty Acids
S.S	Somatostatin
E_2_	Estradiol
PRL	Prolactin
Cor	Cortisol
